# A novel cause of *DKC1*‐related bone marrow failure: Partial deletion of the 3′ untranslated region

**DOI:** 10.1002/jha2.165

**Published:** 2021-01-26

**Authors:** Jonathan W. Arthur, Hilda A. Pickett, Pasquale M. Barbaro, Tatjana Kilo, Raja S. Vasireddy, Traude H. Beilharz, David R. Powell, Emma L. Hackett, Bruce Bennetts, Julie A. Curtin, Kristi Jones, John Christodoulou, Roger R. Reddel, Juliana Teo, Tracy M. Bryan

**Affiliations:** ^1^ Children's Medical Research Institute Faculty of Medicine and Health, University of Sydney Westmead New South Wales Australia; ^2^ Haematology Department Children's Hospital at Westmead Westmead New South Wales Australia; ^3^ Monash Biomedicine Discovery Institute Department of Biochemistry and Molecular Biology, Monash University Clayton Victoria Australia; ^4^ Monash Bioinformatics Platform Monash University Clayton Victoria Australia; ^5^ Department of Molecular Genetics Children's Hospital Westmead Westmead New South Wales Australia; ^6^ Disciplines of Genetic Medicine and Child and Adolescent Health, Faculty of Medicine and Health University of Sydney Westmead New South Wales Australia; ^7^ Department of Clinical Genetics Children's Hospital Westmead Westmead New South Wales Australia; ^8^ Murdoch Children's Research Institute and Department of Paediatrics Melbourne Medical School Parkville Victoria Australia

**Keywords:** DKC1, dyskeratosis congenita, polyadenylation, telomerase, telomeres

## Abstract

Telomere biology disorders (TBDs), including dyskeratosis congenita (DC), are a group of rare inherited diseases characterized by very short telomeres. Mutations in the components of the enzyme telomerase can lead to insufficient telomere maintenance in hematopoietic stem cells, resulting in the bone marrow failure that is characteristic of these disorders. While an increasing number of genes are being linked to TBDs, the causative mutation remains unidentified in 30‐40% of patients with DC. There is therefore a need for whole genome sequencing (WGS) in these families to identify novel genes, or mutations in regulatory regions of known disease‐causing genes. Here we describe a family in which a partial deletion of the 3′ untranslated region (3′ UTR) of *DKC1*, encoding the protein dyskerin, was identified by WGS, despite being missed by whole exome sequencing. The deletion segregated with disease across the family and resulted in reduced levels of *DKC1* mRNA in the proband. We demonstrate that the *DKC1* 3′ UTR contains two polyadenylation signals, both of which were removed by this deletion, likely causing mRNA instability. Consistent with the major function of dyskerin in stabilization of the RNA subunit of telomerase, hTR, the level of hTR was also reduced in the proband, providing a molecular basis for his very short telomeres. This study demonstrates that the terminal region of the 3′ UTR of the *DKC1* gene is essential for gene function and illustrates the importance of analyzing regulatory regions of the genome for molecular diagnosis of inherited disease.

## 1 INTRODUCTION

Telomeres are nucleoprotein complexes capping the ends of chromosomes, composed of tracts of the repeated DNA sequence TTAGGG and sequence‐specific binding proteins. In most human somatic cells, telomeres shorten with each cell division, due to the inability of the DNA replication machinery to replicate the ends of linear DNA molecules; telomere shortening in these cells is a mark of normal aging [1]. In stem cells, including those of the hematopoietic system, the enzyme telomerase counteracts telomere shortening [2]. Telomere biology disorders (TBDs) are a group of rare inherited diseases characterized by the presence of very short telomeres relative to the general population [3, 4, 5, 6]. In these patients, who carry pathogenic variants in components of telomerase or other telomere‐protective proteins, telomeres become abnormally short or dysfunctional, leading to activation of a DNA damage response and cellular senescence or apoptosis. This causes reduced replicative capacity of hematopoietic progenitor cells, leading to progressive bone marrow failure (BMF), a common feature of these disorders and the most common cause of mortality [7].

The first of these diseases linked to short telomeres was dyskeratosis congenita (DC) [4], classically defined by the triad of abnormal skin pigmentation, oral leukoplakia, and nail dystrophy. DC is, however, a multisystem disorder with clinical manifestations that include BMF, pulmonary fibrosis, liver cirrhosis, gastrointestinal symptoms, dental abnormalities, and predisposition to malignancies. BMF is seen in up to 80% of affected patients [7]. Other patients who are now being recognized as having an underlying TBD can present with isolated organ involvement, such as aplastic anemia or pulmonary fibrosis [7].

There are currently 16 genes associated with TBDs, which all play a role in telomerase biogenesis and function, telomere capping, or telomere replication [3, 8, 9]. While an increasing number of genes are being linked with TBDs using single gene analysis, gene panels, or whole exome sequencing (WES), the causative gene remains unidentified in ∼30‐40% of DC patients [10]. There is therefore a need to analyze the genomes of such patients using whole genome sequencing (WGS), to identify novel TBD genes or pathogenic variants in the regulatory regions of known TBD genes.

The X chromosome gene *DKC1*, encoding the protein dyskerin, was the first gene in which pathogenic variants were identified in DC patients [11]. Dyskerin is an RNA‐binding protein that specifically binds and stabilizes the H/ACA family of small RNAs, including the human telomerase RNA subunit (hTR, encoded by the gene *TERC*) [4]. Dyskerin is a pseudouridine synthase and catalyzes the conversion of uridine to pseudouridine at specific sites in ribosomal and spliceosomal RNAs [12]. Dyskerin is also an integral component of the telomerase complex [4, 13, 14]. The discovery that it is responsible for maintaining hTR levels and thereby regulating telomere length, was pivotal in the recognition of the role of telomere biology in the etiology of DC and related disorders [4].

Most patient‐associated variants identified in *DKC1* to date are missense changes, or small deletions or inversions in the protein‐coding region [15, 16], since more extensive deletions are likely to be incompatible with survival [17]. One exception is a family found to harbor a 2 kb deletion, removing the entire last exon of the gene, including the whole 3′ untranslated region (3′ UTR) [18]. Since the terminal 22 amino acids of the protein were also removed, it was unclear whether the protein truncation or loss of the 3′ UTR was responsible for disease.

Here, we describe a family with a much smaller deletion of the 3′ end of the *DKC1* 3′ UTR, that segregates with features of DC across the extended family. This variant was not identified through initial targeted sequencing of DC genes and WES, but was discovered in subsequent WGS analysis. We identified the locations of two polyadenylation signals in the 3′ UTR; this deletion removes both of them, and we demonstrate that this is sufficient to cause dramatic reductions in the levels of *DKC1* mRNA and hTR, which likely leads to the short telomeres of the patient. Thus, we have found that loss of a small portion of the *DKC1* 3′ UTR is sufficient to cause DC. This illustrates the importance of examining the regulatory regions of known disease‐causing genes by WGS in patients for whom a causative variant has yet to be identified.

## 2 METHODS

### 2.1 Subjects

**FIGURE 1:**
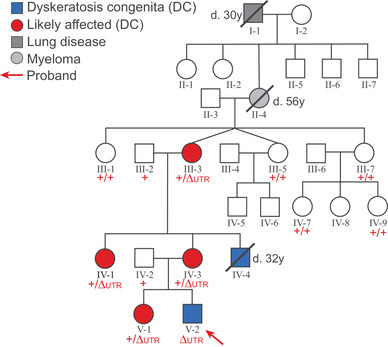
Pedigree of family with dyskeratosis congenita. Relevant phenotypes are shown across five generations. The proband and one maternal uncle have been diagnosed with DC, while several female family members show signs consistent with mild DC (skin pigmentation, dysplastic nails, and premature greying). The genotype at the location of a deletion in the *DKC1* 3′ UTR is shown in red (determined by PCR across the deletion [Figure 2D], and confirmed by Sanger sequencing in individuals IV‐2 and V‐2). ΔUTR indicates the presence of the deletion in Figure 2; + indicates a wild‐type allele

The male proband presented to The Children's Hospital at Westmead at 7 years of age with skin pigmentation, dysplastic nails, dysphagia, and celiac disease, and a family history suggestive of DC (Figure 1, Table 1). Peripheral blood DNA was available from the proband, his parents, sister, maternal grandparents, and 6 other members of the extended family (Figure 1). Informed consent was obtained from all participating individuals, and the studies were approved by the Human Research Ethics Committee of the Sydney Children's Hospitals Network (10/CHW/114).

**TABLE 1:** Clinical features of affected and likely affected family members

	Sex	Age at clinical examination (years)	Hematology	Non‐hematological clinical features
III‐3	F	70	Normal hematology	Dysplastic nails (five) Skin pigmentation (mild)
IV‐1	F	46	Normal hematology	Skin pigmentation (mild)
IV‐3	F	45	Normal hematology	Dysplastic nail (one) Skin pigmentation (mild) Premature greying (from mid‐teens)
IV‐4	M	Died 32 y	Bone marrow failure ‐ thrombocytopenia from adolescence, pancytopenia from early 20s	Dysplastic nails (all) Skin pigmentation Celiac disease Dysphagia Leucoplakia Adermatoglyphia Pulmonary fibrosis Cirrhosis
V‐1	F	20	Normal hematology	Skin pigmentation (extensive)
V‐2 (proband)	M	16	Mild thrombocytopenia from adolescence	Dysplastic nails (all) Skin pigmentation Leucoplakia Celiac disease Mild dysphagia

### 2.2 Genome sequencing and bioinformatic analysis

WGS of peripheral blood genomic DNA from individuals III‐3, IV‐3, V‐1, and V‐2 (Figure 1) was performed on an Illumina HiSeq X Ten platform with 150 bp paired‐end reads, using TruSeq Nano library preparation with 350 bp inserts (Macrogen, Korea). Genome alignment and variant calling were performed by Macrogen using an ISAAC pipeline [19]. The mappable mean read depth was 33‐ to 40‐fold, and 95% of reads were mappable. Variants were analyzed using Ingenuity Variant Analysis (IVA) (Qiagen), first filtering for sequencing quality and confidence by keeping only reads with a call quality of at least 20, and those outside the top 5% most exonically variable 100 base windows in healthy public genomes (1000 Genomes database). Common variants were removed by excluding any with an allele frequency ≥0.5% in either the 1000 Genomes Project, the NHLBI ESP exomes database, or the ExAC database, and only variants present in all four affected individuals were analyzed. A search for all remaining variants in the 14 genes shown to be mutated in TBD patients at the time (*DKC1, TERC, TERT, TINF2, NOP10, NHP2, RTEL1, WRAP53, CTC1, ACD, PARN, STN1, POT1, NAF1^3^*) revealed only a 1104 bp deletion (LRG_55:g.19793_20896del) removing part of the *DKC1* 3′ UTR, and intronic variants in *RTEL1*, *POT1*, and *NAF1*. Outside these 14 genes, there were ∼90 000 variants shared by all four individuals; these were filtered in IVA to keep only missense, frameshift, premature stop codon and splice site changes, insertions and deletions, or variants classified as pathogenic or likely pathogenic, resulting in 159 variants in 116 genes. Manual inspection of these genes did not reveal any with apparent links to telomere biology. We therefore considered the deletion in *DKC1* to be the most likely candidate disease‐causing variant.

### 2.3 PCR across the deletion and Sanger sequencing

**FIGURE 2:**
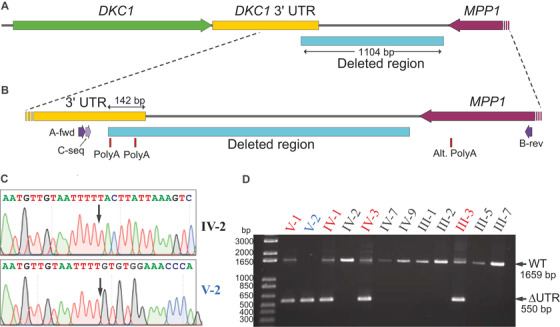
Deletion in the *DKC1* 3′ UTR segregates with disease phenotype. A, Location of the 1104 bp deletion on chromosome X, encompassing part of the *DKC1* 3′ UTR and the intergenic region between genes *DKC1* and *MPP1*. B, Magnification of the deleted region, showing the location of polyadenylation signals (PolyA; confirmed in Figure 4) and primers used for PCR and sequencing (A‐fwd, B‐rev, C‐seq). C, Sanger sequencing (using primer C‐seq) of the PCR product from primers A‐fwd and B‐rev, in the proband (V‐2) and his father (IV‐2). Black arrow marks the 5′ end of the deletion. D, Agarose gel of products of PCR across the deletion in 12 family members. The proband is indicated in blue; shown in red are female family members with signs of disease. The sizes of the wild‐type (WT) or deleted (ΔUTR) PCR products are shown on the right. Note that *DKC1* is on the X chromosome, so male individuals (III‐2, IV‐2, V‐2) carry a single allele

Predesigned PCR primers (Table S1, Figure 2B; ThermoFisher Scientific) included the forward primer of a pair in the *DKC1* 3′ UTR (Hs00402126_CE; here called A‐fwd) and a reverse primer in the *MPP1* gene (Hs00402127_CE; here called B‐rev). PCR reactions included 100 ng genomic DNA, 0.2 µM each primer, 0.2 mM each dNTP, 1.5 mM MgCl_2_, 1 × Platinum Green PCR Buffer (Invitrogen), and 2 U of Platinum *Taq* Green Hot Start DNA Polymerase (Invitrogen). DNA was amplified using 30 cycles of 94°C for 30 seconds, 64°C for 30 seconds, and 72°C for 1 minute. PCR products were electrophoresed at 120 V on a 1% (wt/vol) agarose gel in TBE (89 mM Tris, 89 mM boric acid, 2 mM ethylenediaminetetraacetic acid) and stained in 10 µg/mL ethidium bromide. PCR products sent for Sanger sequencing were purified using a QIAquick PCR Purification kit (Qiagen) and sequenced with primer C‐seq (Figure 2B, Table S1; Sigma‐Aldrich) by AGRF, Westmead, Australia. Sequencing data were visualized and analyzed in SnapGene.

### 2.4 Telomere length analysis (qPCR)

**FIGURE 3:**
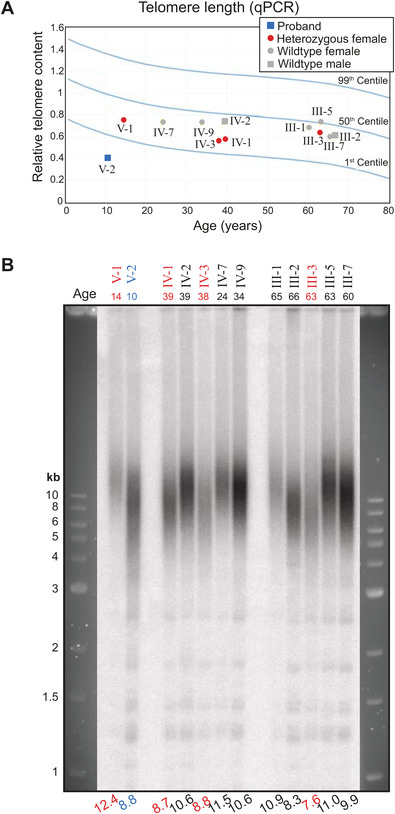
Short telomeres correlate with presence of the *DKC1* deletion. Telomere lengths of 12 family members, measured by qPCR (A) or TRF Southern blot analysis (B). The proband is indicated in blue; shown in red are heterozygous female family members. Curves in (A) represent the indicated percentiles of telomere lengths in ∼240 healthy individuals. Mean TRF lengths in kb are indicated below each lane of the Southern blot in (B)

A previously described monochrome multiplex qPCR telomere length assay [20, 21] was used, in view of its accuracy and sample throughput. Briefly, each reaction was performed on a BioRad CFX386 Touch PCR detection system (BioRad) in 384‐well PCR plates. Twenty nanograms of DNA was added to a master mix containing 300 nM each of *telc* and *telg* telomere PCR primers (Table S1), 350 nM each of *albu* and *albd* single‐copy gene PCR primers and Rotor‐Gene SYBR Green PCR Master Mix (Qiagen), made up to a total volume of 10 µL. Each patient sample was assayed in quadruplicate, and each batch of PCR reactions included four control DNA samples, also assayed in quadruplicate. Telomere content was measured using the ΔΔCT method, using a reference DNA. The difference between CT_alb_ and CT_tel_ was calculated for the reference sample and the control sample. Relative telomere length was then calculated using 2^(CT‐sample – CT‐reference)^. The coefficient of variation (CV) of both the telomere and single copy gene quadruplicate measurements was 1‐2%, while the telomere length CV between batches was 3‐5%. Values obtained from 240 healthy individuals show the normal percentiles for different age groups (Figure 3A).

### 2.5 Patient telomere length analysis (Flow‐FISH)

Telomere flow‐fluorescence in situ hybridization (Flow‐FISH) was performed with a published protocol [22]. Approximately 10 mL of lithium‐heparin peripheral blood was collected, and mononuclear cells were isolated by Ficoll density centrifugation (1077 Ficoll Histopaque, Sigma‐Aldrich). Duplicate samples of 2 × 10^6^ cells were used for FISH analysis. A known cell line with long telomeres (human tetraploid T‐cell lymphoblastic line CCRF‐CEM, GM03671C [22]) served as an internal reference standard for telomere length. Equal numbers of CCRF‐CEM cells were mixed with patient cells prior to hybridization with a fluorescein isothiocyanate‐conjugated (CCCTAA)_3_ peptide nucleic acid probe (Panagene, Korea) at 0.3 µg/mL at 4°C overnight. Flow cytometry was performed on a FACS CANTO II (BD Biosciences, USA) instrument, and data were displayed and analyzed with BD FACSDiva software (BD Biosciences). Calculation of Relative telomere length of the patient's mononuclear cells was performed by comparing the fluorescence of these cells with the tetraploid CCRF‐CEM cell line and expressed as a percentage. Values obtained from 240 healthy individuals show the normal percentiles for different age groups (Figure S1).

### 2.6 Telomere length analysis (southern blot)

Telomere terminal restriction fragments were prepared by *Hin*fI and *Rsa*I digestion of genomic DNA, and 2 µg was loaded on a 1% (wt/vol) agarose gel in 0.5 × TBE. Pulsed‐field gels were run at 6 V/cm for 14 hours at 14°C, with an initial switch time of 1 second and a final switch time of 6 seconds. Gels were dried for 2 hours at 60°C, denatured and hybridized overnight to a [γ‐^32^P]‐ATP‐labeled (CCCTAA)_4_ oligonucleotide probe in Church and Gilbert hybridization buffer [23]. Gels were washed in 4 × SSC (0.06 M sodium citrate, 0.6 M NaCl, pH 7) and exposed to a PhosphorImager screen overnight prior to visualization using a Typhoon TRIO Imager (GE Healthcare Life Sciences). Mean telomere restriction fragment (TRF) lengths (the peak of the smear) were determined by comparison to size markers using ImageQuant TL (GE Healthcare Life Sciences).

### 2.7 Reverse transcription‐PCR (RT‐PCR) of *DKC1* transcripts

RNA was isolated from whole blood from individuals IV‐2, IV‐3, V‐1, and V‐2 using PAXgene Blood RNA Tubes and a PAXgene Blood RNA Kit (PreAnalytiX, Switzerland), following the manufacturer's directions. The 3′ end of *DKC1* mRNA was amplified using the technique of 3′ RACE (Rapid Amplification of cDNA ends). Total RNA (80 ng) was reverse transcribed using oligo(dT)‐based primer AP (Invitrogen; Table S1) and Superscript IV reverse transcriptase (Invitrogen), following the manufacturer's directions. A portion (10%) of the resulting cDNA was amplified by PCR using 0.2 µM primers A‐Fwd and AUAP (Table S1), 0.2 mM each dNTP, 1.5 mM MgCl_2_, 1 × Platinum Green PCR Buffer (Invitrogen), and 2 U of Platinum *Taq* Green Hot Start DNA Polymerase (Invitrogen). DNA was amplified using 30 cycles of 94°C for 30 seconds, 64°C for 30 seconds, and 72°C for 1 minute. PCR products were electrophoresed at 120 V on a 2% (wt/vol) agarose gel in TBE and stained in 10 µg/mL ethidium bromide. A portion (8%) of the PCR products was subjected to nested PCR using the same conditions and primers C‐seq and AUAP (Table S1), and products electrophoresed as above.

### 2.8 Quantitative RT‐PCR analysis (RT‐qPCR)

Total RNA (300 ng) was reverse transcribed as described above, using random hexamers or primer AP for *DKC1* (Table S1), and random hexamer primers (Invitrogen) for *TERC* and *MPP1*. A portion (2.5%) of the cDNA was used for real‐time PCR with Platinum SYBR Green qPCR SuperMix‐UDG (Invitrogen) and 400 nM primers DKFA and DKRA for *DKC1*, hTR‐F and hTR‐R for *TERC*, or a qSTAR qPCR primer pair for *MPP1* (OriGene Technologies; Table S1). PCR was performed in a LightCycler (Roche Life Sciences), incubating at 50°C for 2 minutes and 95°C for 10 minutes, then 40 cycles of 95°C for 15 seconds and 60°C for 1 minute. Expression levels of *DKC1*, *TERC*, and *MPP1* relative to *GAPDH* were calculated using the relative standard curve method in LightCycler software.

### 2.9 Assay for detection of non‐random X‐inactivation

A modified version of the standard HUMARA assay was used to measure X‐chromosome inactivation in female deletion carriers and wild‐type controls [24, 25]. Peripheral blood DNA was digested with restriction enzyme *Dde*I to improve accessibility of methylated regions to PCR, in the presence or absence of methylation‐sensitive enzyme *Hpa*II. Digested and undigested DNA was subjected to PCR using primers across the polymorphic (CAG)_n_ region of the 5′ end of the coding region of the human androgen receptor gene (Met‐F and Met‐R1; Table S1), with the forward primer 5′ end‐labeled with ^32^P. PCR reactions (25 µL) included 80 nM each primer, 0.1 mM each dNTP, 1.5 mM MgCl_2_, 1 × Platinum Green PCR Buffer (Invitrogen), and 2 U of Platinum *Taq* Green Hot Start DNA Polymerase (Invitrogen). DNA was amplified using 19 cycles of 94°C for 30 seconds, 60°C for 30 seconds, and 72°C for 45 seconds. PCR products were electrophoresed at 75 W on a 6% (wt/vol) acrylamide/8 M urea sequencing‐style gel in TBE and exposed to a PhosphorImager screen. Band intensities were quantitated using ImageQuant TL, and the proportion of each allele digested by *Hpa*II (i.e. undermethylated and hence active) was calculated as described [24].

## 3 RESULTS

### 3.1 Identification of deletion of part of *DKC1* 3′ UTR in a family with DC

In this study we performed genetic characterization of a large family presenting with DC (Figure 1). The male proband developed skin pigmentation, dysplastic nails, mild dysphagia, and celiac disease from 7 years of age. The full blood count was normal at diagnosis at 9 years of age, although bone marrow biopsy showed moderately reduced cellularity without morphological or cytogenetic abnormality, in keeping with a failing marrow. Slightly elevated fetal hemoglobin (HbF 1.5‐1.8%) with mild thrombocytopenia with platelet counts of 110‐120 × 10^9^/L developed from 13‐14 years of age. Hemoglobin, leucocyte and neutrophil counts have remained normal, and he remains clinically well at 16 years without specific therapy. A maternal uncle had a similar history, along with leukoplakia and BMF from adolescence, and died from the complications of pulmonary fibrosis and cirrhosis at the age of 32. Several female family members showed skin pigmentation, dysplastic nails, and premature greying but no hematological abnormalities, and the family history included lung disease and myeloma (Figure 1, Table 1). A peripheral blood mononuclear cell sample from the proband showed an average telomere lengthof less than the 1st percentile by Flow‐FISH (Figure S1). Together, these features and family history suggested a diagnosis of DC (OMIM #305000); the milder phenotype of the affected females suggested a possible X‐linked inheritance.

Peripheral blood DNA from the proband was subjected to Sanger sequencing over the entire protein‐coding region and intron‐exon boundaries of the dyskerin (*DKC1)* gene (Centogene GmbH, Germany), and no variants were detected. He was retested using WES, and no significant variants in any of the known DC genes were detected. We therefore performed WGS on peripheral blood DNA from the proband (V‐2), his sister (V‐1), mother (IV‐3), and maternal grandmother (III‐3), to examine structural variants and variants in non‐protein‐coding regions. A 1104 bp deletion encompassing the 3′ end of *DKC1* was observed in all four individuals (Figure 2A). This deletion removed 142 bp at the end of the *DKC1* 3′ UTR, and most of the intergenic region between *DKC1* and the neighboring gene, *MPP1* (Figure 2B). No other non‐intronic variants were detected in the remainder of the *DKC1* gene, or in the other 13 genes known to be mutated in TBD disorders (see Methods section for details).

*DKC1* is an X‐linked gene; the proband (V‐2) is hemizygous for the deletion, whereas the WGS data showed that his mother, sister, and grandmother (IV‐3, V‐1 and III‐3) are heterozygous. The boundaries of the deletion were confirmed by PCR of a region spanning the deletion followed by Sanger sequencing, in the proband and his unaffected father (IV‐2) (Figure 2C). The presence or absence of the deletion was determined by PCR in 12 members of the extended family (Figure 2D). All female family members displaying skin, nail, and hair symptoms (labeled in red in Figure 2D) are heterozygous for the deleted allele, whereas no asymptomatic individual carries the deletion. The 3′ UTR deletion in *DKC1* therefore segregates perfectly with disease across this large family.

### 3.2 Carriers of the deletion have moderately short telomeres

To provide additional evidence for the link between the 3′ UTR *DKC1* deletion and telomere‐related disease, we measured telomere lengths of the 12 members of the extended family using quantitative PCR (Figure 3A). In agreement with the Flow‐FISH result (Figure S1), telomere length in the proband was well below the 1st centile of the normal population (Figure 3A). Southern blot analysis of TRFs also showed that the shortest telomere fragments of the proband (V‐2) were shorter than those of any of his relatives, despite his young age (Figure 3B). Heterozygous female carriers of the deletion (red, Figures 3A and 3B, Figure S1) had telomeres that were comparable to or slightly shorter than those of wild‐type individuals of similar age by qPCR, TRF, and Flow‐FISH analysis, consistent with their mild DC symptoms. Thus, telomere lengths of the extended family are consistent with the *DKC1* deletion being causative of disease.

### 3.3 The proband expresses very low levels of *DKC1* mRNA

**FIGURE 4:**
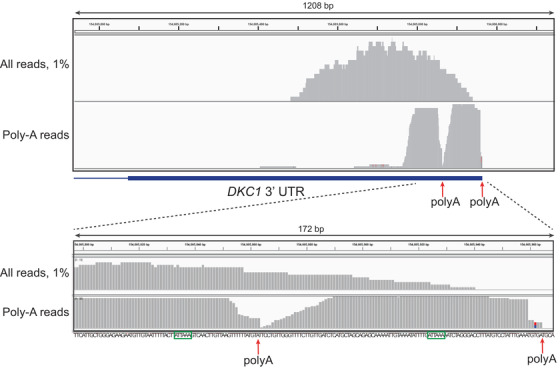
Location of polyadenylation sites in 3′ UTR of *DKC1* gene. Single‐cell RNA sequencing data from peripheral blood mononuclear cells from a healthy donor [28] were sub‐sampled to identify RNAseq reads in the *DKC1* 3′ UTR. The top panel represents the whole 3′ UTR (thick blue line), while the bottom panel shows a zoomed‐in representation of the area around the polyadenylation signals. Sequencing tracts represent 1% of all reads mapping to the 3′ UTR (top) or all reads extending into non‐templated poly(A) tracts (bottom). There are two peaks of reads containing poly(A) tracts; the right edge of each peak is the site of polyadenylation (red arrows). The bottom panel includes the sequence of this region of the 3′ UTR, with the two polyadenylation signals (ATTAAA) outlined in green

Functional analysis was then performed to determine the impact of the deletion on the function of dyskerin. The 3′ UTRs of genes are involved in many gene regulatory processes, including transcript polyadenylation and stability, translation efficiency, and microRNA binding [26]. Polyadenylation of mammalian mRNAs occurs 10‐30 nt downstream of a conserved polyadenylation signal of sequence AAUAAA or AUUAAA [27]. Inspection of the *DKC1* 3′ sequence revealed two AUUAAA motifs, 132 nt and 41 nt upstream of the end of the transcript, respectively (Figure 2B). To determine whether either or both of these sequences constitute the poly(A) signal of *DKC1*, we examined a published single‐cell transcriptomic dataset from human peripheral blood mononuclear cells [28], where the 3′‐focused sequencing reads extended into non‐templated poly(A) stretches that correspond to polyadenylation. For the *DKC1* locus, we saw two peaks of such reads associated with each of the AUUAAA motifs (Figure 4). Thus, *DKC1* is expressed in mononuclear cells as two 3′ UTR isoforms. The deleted allele of *DKC1* is lacking both of the canonical poly(A) signals, and hence the stability of transcripts arising from this allele is likely to be compromised.

**FIGURE 5:**
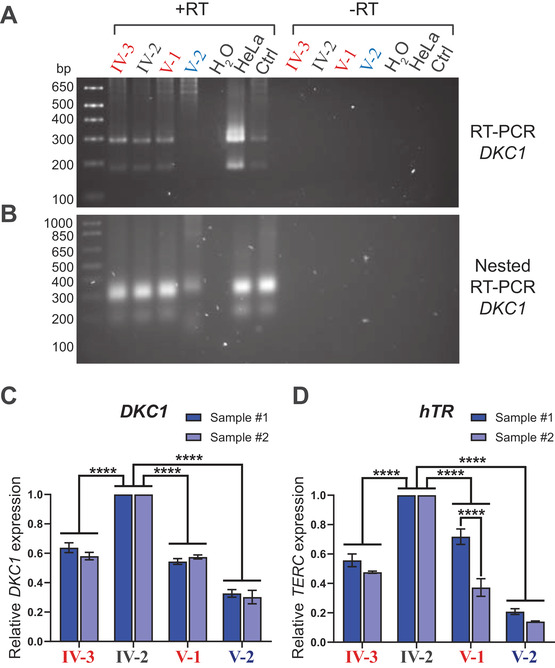
The proband expresses very low levels of *DKC1* mRNA and telomerase RNA. A, RT‐PCR of *DKC1* transcripts in peripheral blood cells of the proband (V‐2) and his immediate family members, and RNA from HeLa cells or an unrelated individual (Ctrl) as wild‐type controls. −RT: negative control in the absence of reverse transcriptase. B, A second round of nested PCR was performed on the products from (A), using an internal primer. C, Levels of *DKC1* mRNA, measured by quantitative real‐time RT‐PCR (RT‐qPCR) using random hexamers for cDNA transcription in the proband (V‐2) and his immediate family members. Female relatives heterozygous for the deletion are labeled in red. Data shown as mean ± SEM of expression levels relative to individual IV‐2; ****P < .0001, as determined by two‐way ANOVA followed by Tukey's multiple comparison tests; n = 3‐4 independent RT reactions from each of two blood samples. D, Levels of the telomerase RNA subunit, hTR, measured by RT‐qPCR using random hexamers for cDNA transcription in the proband (V‐2) and his immediate family members. Female relatives heterozygous for the deletion are labeled in red. Data shown as mean ± SEM of expression levels relative to individual IV‐2; ****P < .0001, as determined by two‐way ANOVA followed by Tukey's multiple comparison tests; n = 5 independent RT reactions from each of two blood samples

To determine whether polyadenylated *DKC1* transcripts from the deleted allele were detectable, 3′ RACE (Rapid Amplification of cDNA ends) was performed using a tailed oligo(dT) primer for reverse transcription, followed by PCR with the tail primer and a primer within the *DKC1* 3′ UTR (A‐fwd; Figure 2B). RACE was performed on RNA isolated from peripheral blood of the proband, his parents, and sister, and products analyzed by gel electrophoresis (Figure 5A). Transcription of the wild‐type or deleted alleles would result in products of 283 bp and 141 bp, respectively. A band consistent with the size of the wild‐type allele was seen in all wild‐type and heterozygous individuals and controls, along with an additional band of ∼180 bp consistent with a shorter transcript utilizing the internal poly(A) signal. No bands in this size range were detected in the RACE products from the proband (Figure 5A). To increase specificity and sensitivity of the PCR, the products were subjected to an additional round of PCR using a “nested” gene‐specific primer (C‐seq; Figure 2B). The same two bands were observed in heterozygous and wild‐type individuals, and faint bands of different sizes were detected in the proband (Figure 5B). These data suggest that RNA transcribed from the deleted allele of *DKC1* may be utilizing an alternative poly(A) signal, such as one present on the non‐transcribed strand of the 3′ end of the neighboring *MPP1* gene (Figure 2B) [18], and this results in an unstable transcript.

To more quantitatively determine the level of *DKC1* mRNA in peripheral blood cells from the proband, the cDNA from the same four family members was subjected to quantitative real‐time reverse transcription‐PCR (RT‐qPCR) using primers within the protein‐coding region of *DKC1*. The proband had levels of *DKC1* mRNA ∼30% of those of his wild‐type father, whereas his heterozygous mother and sister had intermediate levels of *DKC1* transcripts (Figure 5C). Similar results were observed using cDNA reverse transcribed with either random hexamers (Figure 5C) or an oligo(dT) primer (Figure S2A), indicating that a majority of the proband's *DKC1* transcripts were likely to be polyadenylated through use of an alternative poly(A) signal. It has been reported that blood cells of most female carriers of X‐linked DC show skewed X‐chromosome inactivation, with ≥95% transcription arising from their WT allele [29, 30, 31]. We measured the degree of skewed X‐inactivation in peripheral blood of three of the female carriers using a standard assay involving digestion with a methylation‐sensitive restriction enzyme followed by PCR across a heterozygous region of the androgen receptor gene on the X‐chromosome (Figure S3). Carriers of the mutation showed substantially skewed inactivation of a single allele (89‐96%), consistent with previous studies, whereas in the two wild‐type females, the two alleles were approximately equally likely to be inactivated. The reduced *DKC1* expression observed in the two female carriers analyzed here, relative to a WT male, is therefore likely to be only partly due to the reduced stability of the mutant transcript. This is consistent with their almost‐normal telomere lengths and very mild presentation of DC features.

Since one end of the deletion was very close to the 3′ UTR of the neighboring gene, *MPP1* (Figure 2), we also measured levels of expression of *MPP1* in the same four family members by RT‐qPCR. *MPP1* transcript levels varied between individuals and between different blood samples from each individual, but did not correlate with presence or absence of the *DKC1* deletion (Figure S2B). We therefore conclude that the deletion in this family does not affect *MPP1* expression.

### 3.4 The proband has low levels of telomerase RNA

One of the major functions of dyskerin is to stabilize the RNA subunit of telomerase (hTR) [4, 32], so levels of hTR in peripheral blood RNA were determined by RT‐qPCR in these four family members. Again, the proband had much lower hTR levels (∼20%) than his father; his mother and sister had intermediate hTR levels (Figure 5D).

## 4 DISCUSSION

Most patient‐associated variants in *DKC1* are missense changes, or small deletions or inversions in the coding region [15, 16]. We describe here the first example of a family with DC who instead carries a deletion of a small region of the *DKC1* 3′ UTR. Multiple lines of evidence support the pathogenicity of this deletion: (a) the deletion segregates with disease and correlates with t across a large family, (b) steady‐state levels of *DKC1* mRNA are greatly reduced in peripheral blood cells from the proband, and (c) the proband also has dramatically reduced levels of the telomerase RNA subunit, hTR. Past studies have shown that a 50‐60% reduction of hTR levels in peripheral blood cells or fibroblasts from *DKC1*‐mutated DC patients is sufficient to lead to telomere shortening and disease [33, 34]; the 80% reduction in hTR observed in the patient in this study therefore provides a molecular explanation for his short telomeres.

Discovery of this deletion illustrates that the last 142 nt of the 822 nt *DKC1* 3′ UTR are essential for full stability of the *DKC1* mRNA. This is most likely because this region contains both canonical polyadenylation signals for *DKC1*; polyadenylation is known to promote mRNA stability by providing a binding platform for proteins and protecting against exonucleolytic degradation [35]. Nevertheless, RT‐PCR using an oligo(dT) primer indicated that some poly(A)‐containing *DKC1* transcript exists in the proband's blood cells, albeit at greatly reduced levels (Figure 5B, Figure S2A). The size of this transcript is consistent with use of a cryptic polyadenylation signal in the antisense strand of the neighboring *MPP1* gene, as has previously been demonstrated in a patient missing the whole *DKC1* 3′ UTR [18]. The existence of this cryptic polyadenylation signal is likely the only reason that a *DKC1* 3′ UTR deletion is compatible with survival in male patients, who are necessarily hemizygous for their *DKC1* variant, since complete loss of dyskerin expression is known to be lethal in mice [17]. The identification of the two canonical polyadenylation signals in *DKC1* may have implications for future therapy of patients with deletions in this region; with emerging gene editing technologies it might become possible to engineer a more effective polyadenylation signal upstream of the deletion, resulting in a higher level of *DKC1* expression than is conferred by the existing cryptic polyadenylation signal.

Genomic DNA from the proband was initially analyzed by targeted sequencing of the protein‐coding region of *DKC1*, as well as by WES, without this deletion being detected. WES has the potential to detect deletions or other variants in UTRs, since UTRs are within exons; however, the kits currently used for enriching for exonic regions of DNA vary in which regions of the genome they target and their ability to capture UTRs [36]. This DC family is therefore an excellent example of the importance of thorough analysis of non‐coding regions of the genome using WGS or targeted gene sequencing for molecular diagnosis of inherited disease.

## CONFLICT OF INTEREST

The authors declare that there is no conflict of interest that could be perceived as prejudicing the impartiality of the research reported.

## AUTHOR CONTRIBUTIONS

Jonathan W. Arthur performed bioinformatic analysis and identified the deletion in *DKC1*. Juliana Teo and Kristi Jones collected clinical data, samples, and patient consent. Hilda A. Pickett, Pasquale M. Barbaro, Tatjana Kilo, Raja S. Vasireddy, and Julie A. Curtin performed and analyzed patient telomere length analysis. Traude H. Beilharz and David R. Powell identified the polyadenylation sites. Emma L. Hackett and Bruce Bennetts confirmed the deletion in a clinically‐accredited laboratory. Tracy M. Bryan, John Christodoulou, Roger R. Reddel, and Juliana Teo designed the study. Tracy M. Bryan performed all remaining experiments and wrote the manuscript, and all of the authors edited the manuscript.

## Supporting information

Supporting information

## Data Availability

All data that support the findings of this study are available from the corresponding author upon reasonable request.
