# Supplementary material for: A novel cause of DKC1‐related bone marrow failure: Partial deletion of the 3′ untranslated region
Source: EJHaem. 2021 Jan 26;2(2):157–66. doi: 10.1002/jha2.165 (PMC9175968; doi:10.1002/jha2.165)
Supplement: Supplementary file 1 — Supporting information [file JHA2-2-157-s001.pdf]

SUPPLEMENTAL MATERIAL

A novel cause of *DKC1*-related bone marrow failure: partial deletion of the 3' untranslated region

Jonathan W. Arthur, Hilda A. Pickett, Pasquale M. Barbaro, Tatjana Kilo, Raja S. Vasireddy, Traude H. Beilharz, David R. Powell, Emma L. Hackett, Bruce Bennetts, Julie A. Curtin, Kristi Jones, John Christodoulou, Roger R. Reddel, Juliana Teo and Tracy M. Bryan

This supplemental document includes:

Supplemental Table 1: DNA oligonucleotides used in this study

Supplemental Figure 1: Flow-FISH analysis of telomere length in peripheral blood mononuclear cells of proband and a subset of his relatives.

Supplemental Figure 2: The proband expresses very low levels of polyadenylated *DKC1* mRNA, but *MPP1* transcript levels do not correlate with presence of the deletion.

Supplemental Figure 3: Skewed X-inactivation in female carriers of the *DKC1* deletion.

Supplemental Table 1. DNA oligonucleotides used in this study.

Primer Name	Sequence (5'-3')
**A-fwd**	GCTCACCTAAATCCATCTGACTACT
**B-rev**	AACAGGGCTGCTCCAACTAGTTTT
**C-seq**	GTTCCTGTGCCCTCTTGTTT
**telc**	TGTTAGGTATCCCTATCCCTATCCCTATCCCTATCCCTAACA
**telg**	ACACTAAGGTTTGGGTTTGGGTTTGGGTTTGGGTTAGTGT
**albu**	CGGCGGCGGGCGGCGCGGGCTGGGCGGAAATGCTGCACAGAATCCTTG
**albd**	GCCCGGCCCGCCGCGCCCGTCCCGCCGGAAAAGCATGGTCGCCTGTT
**Adapter primer (AP)**	GGCCACGCGTCGACTAGTACTTTTTTTTTTTTTTTTT
**Abridged Universal Adapter Primer (AUAP)**	GGCCACGCGTCGACTAGTAC
**DKFA**	GCCGAAATACAACACGCTGAAG
**DKRA**	CAGAGGATTTGAACCACATGCA
**hTR-F**	CTAACCCTAACTGAGAAGGGCGTA
**hTR-R**	GGCGAACGGGCCAGCAGCTGACATT
**MPP1-F**	CTCTACCTGGAGCATTTGCTGC
**MPP1-R**	GTATGAGTCGCACTTTCCGCAC
**GAPDH-F**	ACCCACTCCTCCACCTTTG
**GAPDH-R**	CTCTTGTGCTCTTGCTGGG
**Met-F**	TCCAGAATCTGTTCCAGAGCGTGC
**Met-R1**	GCTGTGAAGGTTGCTGTTCCTCAT

Supplemental Figure 1

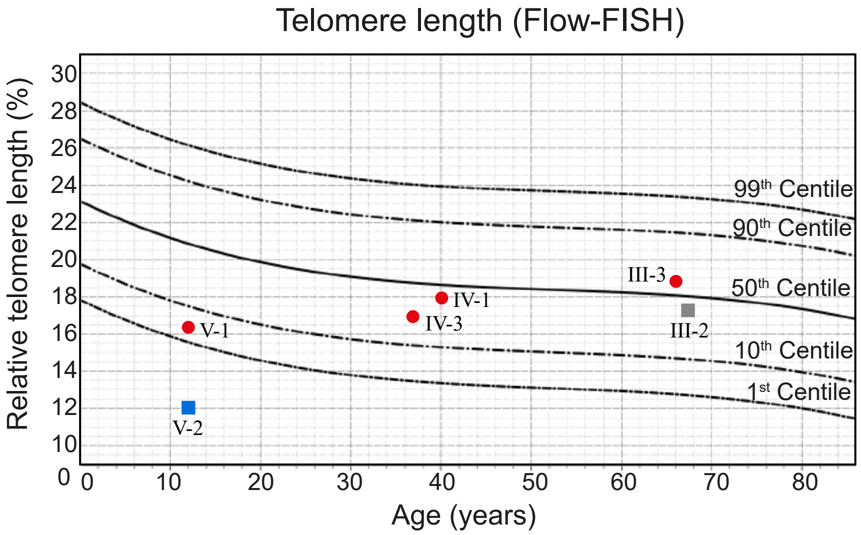

Supplemental Figure 1: Flow-FISH analysis of telomere length in peripheral blood mononuclear cells.

Telomere lengths of the proband (blue) and a subset of his relatives, measured by Flow-FISH. Heterozygous females shown in red; wild-type grandfather shown in grey. Curves represent the indicated percentiles of telomere lengths in ~240 healthy individuals.

Supplemental Figure 2

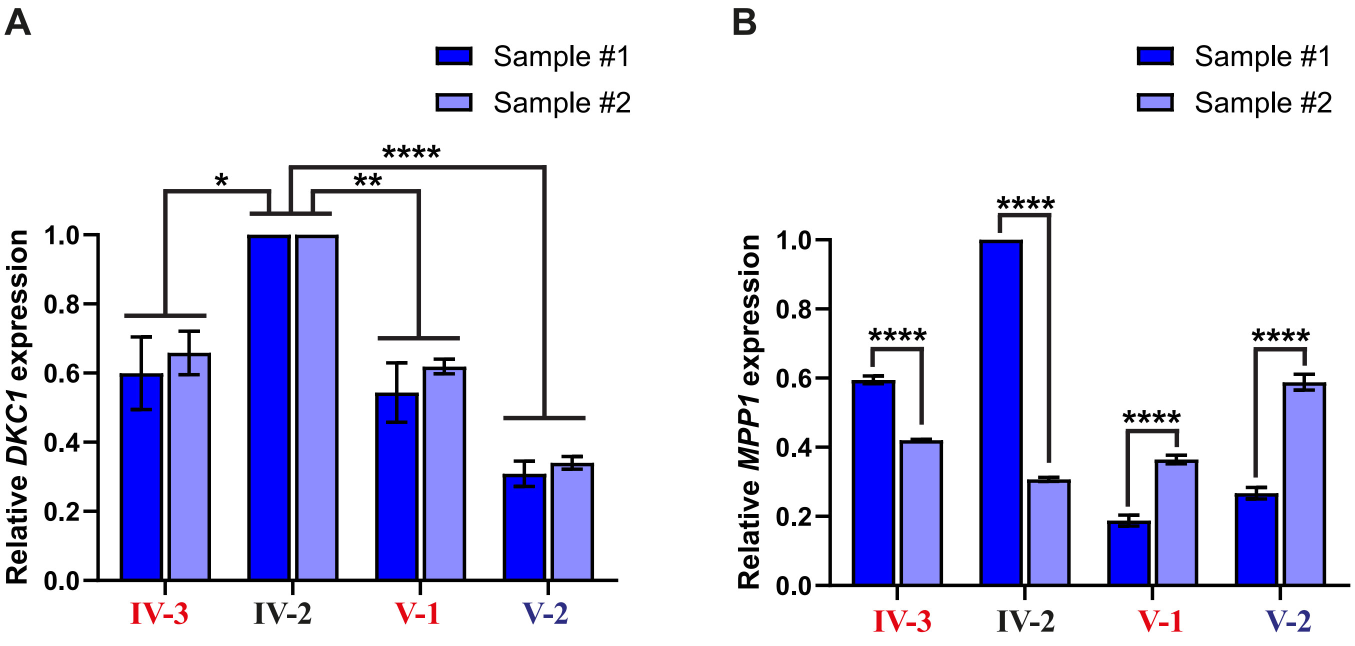

Supplemental Figure 2: The proband expresses very low levels of polyadenylated *DKC1* mRNA, but *MPP1* transcript levels do not correlate with presence of the deletion.

(A) Levels of *DKC1* mRNA, measured by quantitative real-time RT-PCR (qRT-PCR) using an oligo(dT) primer for cDNA transcription in the proband (V-2) and his immediate family members. Female relatives heterozygous for the deletion are labeled in red. Data shown as mean ± SEM of expression levels relative to individual IV-2; *$p < 0.05$, **$p < 0.01$, ****$p < 0.0001$, determined by 2-way ANOVA followed by Tukey's multiple comparison tests; n = 3 independent RT reactions from each of two blood samples. (B) Levels of *MPP1* mRNA, measured by qRT-PCR using random hexamers for cDNA transcription in the proband (V-2) and his immediate family members. Female relatives heterozygous for the deletion are labeled in red. Data shown as mean ± SEM of expression levels relative to individual IV-2; ****$p < 0.0001$, determined by 2-way ANOVA followed by Tukey's multiple comparison tests; n = 3 independent RT reactions from each of two blood samples.

Supplemental Figure 3

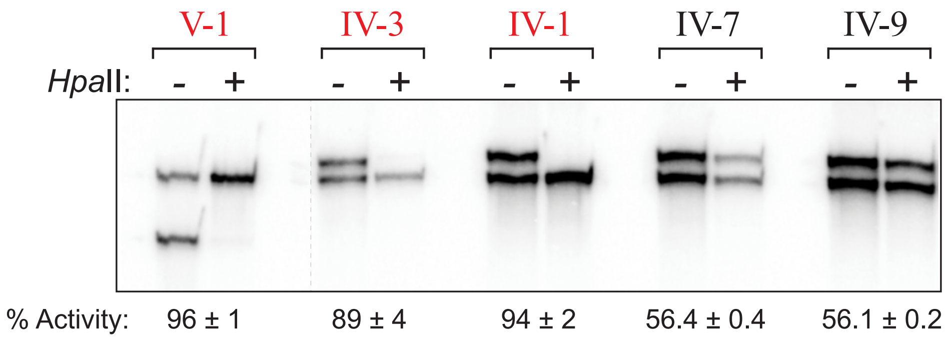

Supplemental Figure 3: Skewed X-inactivation in female carriers of the *DKC1* deletion.

A PCR-based assay was used to distinguish between the two X-chromosomes of the indicated female subjects, and to determine the percentage of transcriptional activity of each chromosome. Peripheral blood genomic DNA was digested with restriction enzyme *Hpa*II, which only cleaves undermethylated (i.e. transcriptionally active) DNA. PCR was performed across the highly polymorphic and therefore typically heterozygous $(CAG)_n$ region of the 5′ end of the coding region of the human androgen receptor gene (HUMARA) at chromosome Xq11.2; the active allele will be cleaved with *Hpa*II and therefore cannot be PCR amplified. Females heterozygous for the deletion are shown in red; subject III-3 was not included because she was homozygous at this locus and therefore uninformative. Numbers beneath the gel indicate the percentage of the most active allele in each individual; mean ± SEM of 3 independent PCR reactions.
